# Supplementary material for: Withania somnifera L.: Phenolic Compounds Composition and Biological Activity of Commercial Samples and Its Aqueous and Hydromethanolic Extracts
Source: Antioxidants (Basel). 2023 Feb 22;12(3):550. doi: 10.3390/antiox12030550 (PMC10045402; doi:10.3390/antiox12030550)
Supplement: Supplementary file 1 [file antioxidants-12-00550-s001.zip › antioxidants-2204029-supplementary.pdf]

Table S1. Acetylcholinesterase (AChE) and butyrylcholinesterase (BChE) activity (% comparing to control samples) after treatment by 9-Amino-1,2,3,4-tetrahydroacridine hydrochloride hydrate

Concentration	0 μM	10 μM	15 μM	20 μM	25 μM	50 μM
AChE inhibition%	100	32.1	23.6	19.4	15.0	9.0
BChE inhibition%	100	17.2	11.3	9.0	7.7	4.0

Table S2. Pearson's correlation analysis for the hydromethanolic extracts of the tested *W. somnifera* commercial samples.

	TPC	TF	TPA	ASA	DPPH	ABTS	FRAP	GA	CAT	VA	CA	FA	SYN	pCA	RUT	Q	NAR	AChE	BChE
TPC	1.000																		
TF	0.222	1.000																	
TPA	**0.531**	0.433	1.000																
ASA	**0.486**	**0.520**	**0.746**	1.000															
DPPH	-0.189	**-0.474**	**-0.482**	**-0.672**	1.000														
ABTS	**0.582**	0.291	0.881	**0.787**	-0.351	1.000													
FRAP	0.446	**0.740**	**0.564**	**0.505**	-0.333	**0.537**	1.000												
GA	-0.097	0.415	**0.513**	**0.555**	**-0.483**	0.394	0.323	1.000											
CAT	0.234	**0.863**	0.368	0.380	-0.381	0.226	**0.871**	0.439	1.000										
VA	0.261	-0.094	0.010	0.215	-0.150	0.142	-0.261	-0.342	-0.320	1.000									
CA	0.440	**0.622**	**0.567**	0.289	-0.281	0.447	**0.849**	0.177	**0.734**	-0.272	1.000								
FA	-0.095	0.212	0.093	-0.152	-0.133	-0.155	0.288	0.407	**0.488**	-0.465	0.274	1.000							
SYN	**0.689**	**0.513**	**0.682**	**0.804**	-0.357	**0.769**	**0.683**	**0.528**	**0.536**	0.030	0.426	0.061	1.000						
pCA	0.405	**0.613**	**0.780**	**0.800**	**-0.623**	**0.679**	**0.659**	**0.803**	**0.643**	-0.206	**0.573**	0.268	**0.783**	1.000					
RUT	**0.489**	0.133	0.169	0.259	**-0.533**	0.140	0.283	0.147	0.260	0.150	0.201	0.359	0.404	0.304	1.000				
Q	-0.116	0.409	0.042	-0.122	0.031	0.009	**0.640**	-0.026	**0.610**	**-0.473**	**0.743**	0.231	-0.039	0.157	-0.158	1.000			
NAR	0.465	0.128	0.250	0.273	**-0.560**	0.156	0.227	0.171	0.202	0.120	0.179	0.360	0.343	0.314	**0.961**	-0.211	1.000		
AChE	0.185	-0.289	-0.098	0.162	-0.002	0.039	**-0.552**	-0.145	**-0.591**	**0.556**	**-0.546**	**-0.514**	0.002	-0.141	0.018	**-0.725**	0.039	1.000	
BChE	-0.105	-0.127	-0.127	0.110	-0.347	-0.134	-0.403	0.167	-0.221	0.307	-0.282	-0.132	-0.147	0.136	0.106	-0.337	0.122	**0.562**	1.000

Statistically significant correlations ($p < 0.05$) are in bold.

TPC: total phenolic compounds, TF: total flavonoids, TPA: total phenolic acids, ASA: L(+) ascorbic acid, DPPH: 2,2-diphenyl-1-picryl-hydrazyl assay, ABTS: 2,2'-azinobis-(3-ethylbenzothiazoline-6-sulfonate) assay, FRAP: ferric reducing antioxidant power assay, GA-gallic acid, CAT-catechin, VA-vanilic acid, CA-caffeic acid, pCA-p-coumaric acid, FA-ferulic acid, SYN – sinapinic acid, RUT-rutin, Q-quercetin, NAR- naringenin, AChE: acetylcholinesterase inhibitory activity, BChE: butyrylcholinesterase inhibitory activity.

Table S3. Pearson's correlation analysis for the aqueous extracts of the tested *W. somnifera* commercial samples.

	TPC	TF	TPA	ASA	DPPH	ABTS	FRAP	GA	CAT	VA	CA	FA	SYN	pCA	RUT	Q	NAR	AChE	BChE
TPC	1.000																		
TF	0.286	1.000																	
TPA	**0.635**	-0.061	1.000																
ASA	0.113	-0.252	0.293	1.000															
DPPH	0.222	0.318	-0.212	0.021	1.000														
ABTS	0.445	0.075	**0.565**	-0.214	-0.114	1.000													
FRAP	0.401	-0.461	**0.591**	0.254	0.039	0.289	1.000												
GA	0.286	0.092	0.436	0.211	-0.121	0.199	0.321	1.000											
CAT	**0.628**	-0.128	**0.720**	0.375	-0.137	0.418	**0.783**	**0.482**	1.000										
VA	**0.622**	0.201	**0.623**	0.319	0.466	0.335	0.371	0.018	0.396	1.000									
CA	0.404	0.069	**0.588**	**0.592**	0.013	0.110	0.256	**0.533**	0.450	**0.577**	1.000								
FA	**0.599**	0.136	**0.690**	0.284	-0.144	0.231	0.404	**0.653**	**0.654**	0.287	**0.590**	1.000							
SYN	0.167	0.404	-0.067	0.256	**0.625**	-0.055	0.176	-0.062	0.221	0.425	0.079	-0.004	1.000						
pCA	**0.743**	0.348	**0.692**	0.166	-0.139	**0.480**	0.197	0.415	**0.635**	**0.499**	**0.537**	**0.595**	0.046	1.000					
RUT	**0.672**	-0.039	**0.824**	0.249	-0.083	0.424	0.464	0.172	**0.521**	**0.670**	**0.614**	**0.639**	-0.002	**0.611**	1.000				
Q	-0.176	**0.588**	**-0.637**	-0.408	0.345	-0.329	**-0.614**	-0.145	**-0.611**	-0.286	-0.226	-0.220	0.046	-0.350	-0.448	1.000			
NAR	-0.201	0.079	-0.115	-0.062	-0.304	0.036	-0.103	-0.014	-0.195	-0.229	-0.042	-0.059	-0.151	-0.059	-0.035	0.238	1.000		
AChE	-0.234	-0.432	0.178	0.123	-0.376	0.269	0.031	**-0.506**	-0.015	0.025	-0.139	-0.145	-0.076	-0.121	0.286	**-0.475**	0.011	1.000	
BChE	0.263	0.045	0.216	0.372	0.140	0.193	-0.127	-0.175	0.134	**0.469**	0.164	0.013	0.246	0.442	0.191	-0.397	-0.234	0.290	1.000

Statistically significant correlations ($p < 0.05$) are in bold

TPC: total phenolic compounds, TF: total flavonoids, TPA: total phenolic acids, ASA: L(+) ascorbic acid, DPPH: 2,2-diphenyl-1-picryl-hydrazyl assay, ABTS: 2,2'-azinobis-(3-ethylbenzothiazoline-6-sulfonate) assay, FRAP: ferric reducing antioxidant power assay, GA-gallic acid, CAT-catechin, VA-vanilic acid, CA-caffeic acid, pCA-p-coumaric acid, FA-ferulic acid, SYN – sinapinic acid, RUT-rutin, Q-quercetin, NAR- naringenin, AChE: acetylcholinesterase inhibitory activity, BChE: butyrylcholinesterase inhibitory activity.
